# *ATF3* characterizes aggressive drug-tolerant persister cells in HGSOC

**DOI:** 10.1038/s41419-024-06674-x

**Published:** 2024-04-24

**Authors:** Kathrin Böpple, Yaara Oren, Whitney S. Henry, Meng Dong, Sandra Weller, Julia Thiel, Markus Kleih, Andrea Gaißler, Damaris Zipperer, Hans-Georg Kopp, Yael Aylon, Moshe Oren, Frank Essmann, Chunguang Liang, Walter E. Aulitzky

**Affiliations:** 1 Dr. Margarete Fischer-Bosch - Institute of Clinical Pharmacology and University of Tuebingen, Auerbachstr. 112, 70376 Stuttgart, Germany; 2 Department of Human Molecular Genetics & Biochemistry, Faculty of Medicine, Tel Aviv University, Tel Aviv-Yafo, Israel; 3 Whitehead Institute for Biomedical Research, 455 Main St., Cambridge, MA 02142 USA; 4 Robert Bosch Hospital, Auerbachstr. 110, 70376 Stuttgart, Germany; 5 Robert Bosch Center for Tumor Diseases (RBCT), Auerbachstr. 110, 70376 Stuttgart, Germany; 6 Weizmann Institute of Science, 234 Herzl St, Rehovot, Israel; 7 Department of Bioinformatics, Biocenter Am Hubland, University of Wuerzburg, 97074 Wuerzburg, Germany; 8 Institute of Immunology, Jena University Hospital, Friedrich-Schiller-University, Leutragraben 3, 07743 Jena, Germany; 9 Present Address: Robert Bosch Center for Tumor Diseases (RBCT), Auerbachstr. 110, 70376 Stuttgart, Germany

**Keywords:** Cancer models, Ovarian cancer, Tumour heterogeneity

## Abstract

High-grade serous ovarian cancer (HGSOC) represents the most common and lethal subtype of ovarian cancer. Despite initial response to platinum-based standard therapy, patients commonly suffer from relapse that likely originates from drug-tolerant persister (DTP) cells. We generated isogenic clones of treatment-naïve and cisplatin-tolerant persister HGSOC cells. In addition, single-cell RNA sequencing of barcoded cells was performed in a xenograft model with HGSOC cell lines after platinum-based therapy. Published single-cell RNA-sequencing data from neo-adjuvant and non-treated HGSOC patients and patient data from TCGA were analyzed. DTP-derived cells exhibited morphological alterations and upregulation of epithelial-mesenchymal transition (EMT) markers. An aggressive subpopulation of DTP-derived cells showed high expression of the stress marker *ATF3*. Knockdown of *ATF3* enhanced the sensitivity of aggressive DTP-derived cells to cisplatin-induced cell death, implying a role for *ATF3* stress response in promoting a drug tolerant persister cell state. Furthermore, single cell lineage tracing to detect transcriptional changes in a HGSOC cell line-derived xenograft relapse model showed that cells derived from relapsed solid tumors express increased levels of EMT and multiple endoplasmic reticulum (ER) stress markers, including *ATF3*. Single cell RNA sequencing of epithelial cells from four HGSOC patients also identified a small cell population resembling DTP cells in all samples. Moreover, analysis of TCGA data from 259 HGSOC patients revealed a significant progression-free survival advantage for patients with low expression of the *ATF3*-associated partial EMT genes. These findings suggest that increased *ATF3* expression together with partial EMT promote the development of aggressive DTP, and thereby relapse in HGSOC patients.

## Introduction

High grade serous ovarian carcinoma (HGSOC), the most frequent histological subtype of ovarian cancer, is one of the deadliest cancers for women [1]. Most HGSOC patients initially respond to chemotherapy. However, relapse commonly occurs and a gradual loss of sensitivity to treatment is associated with each recurrence [2]. This indicates that a fraction of cancer cells persist most likely due to transient activation of non-genetic protective programs. In vitro, “drug-tolerant persister” (DTP) cells have been described in various cancer types as a cell subpopulation that survives initial cancer treatment without the development of permanent resistance via de novo gene mutations [3]. It is still uncertain whether these cells pre-exist in the population or cells enter this state upon exposure to the treatment [4]. The principles underlying the phenotype of cancer cell persistence in HGSOC are poorly understood [5]. Conceivably, minimal residual disease consisting of DTP cells may play a major role in the evolution towards highly aggressive, non-responsive and finally lethal HGSOC. Identifying the mechanisms that underlie tumor evolution and promote drug tolerance is one of the critical challenges in ovarian cancer research [6]. Because persistence is expected to develop more rapidly than resistance, it is particularly important to identify the transcriptional and translational programs enabling cancer cells to survive cytotoxic treatment.

Several mechanisms were described that contribute to cancer cell persistence. One of those is cell identity change via epithelial-mesenchymal transition (EMT) [7]. EMT is an early event prior to metastasizing or evading anti-cancer treatment [8]. The cancer cells undergoing the EMT process adopt a spindle-shaped cell morphology, increased motility and gain invasive properties [9]. In addition, they downregulate epithelial markers such as EPCAM and E-cadherin and upregulate mesenchymal markers such as Vimentin, Snail and Twist [10–12].

Rather little is known regarding the mechanisms underlying persister cell development in HGSOC. To identify such strategies, we generated a cellular model system of DTP-derived HGSOC cells, revealing elevated expression of *ATF3*-associated partial EMT markers in DTP. In addition, a subset of highly aggressive DTP cells exhibited increased expression of stress response genes. Similarly, enhanced expression of EMT and stress response genes was observed in a CDX model of HGSOC relapse cells after platinum-based therapy. Importantly, the existence of such subpopulation was confirmed also in scRNA-seq datasets from 4 HGSOC patients.

## Results

### Functional and morphological alterations of drug tolerant persister cells

**Fig. 1:**
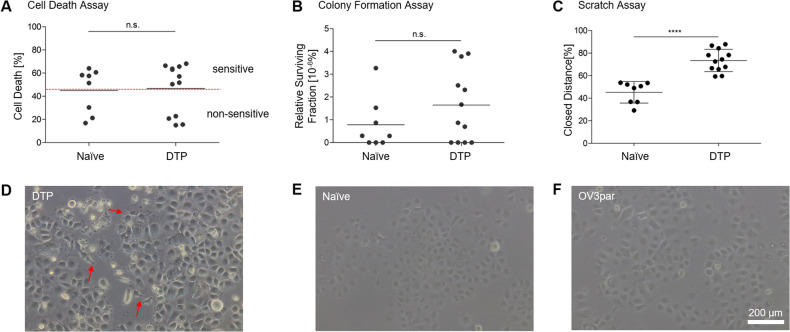
Phenotypic alterations distinguish drug tolerant persister cells (DTPs) from treatment-naïve cells. **A** Treatment-naïve (Naïve) and DTP-derived (DTP) cell clones were treated with cisplatin for 48 h and cell death (Annexin V/PI) was analyzed by flow cytometry. Cell clones were categorized as sensitive or non-sensitive relative to the combined average of cell death in control and DTPs cell clones. **B** Colony formation assays of naïve and DTP cell clones after exposure to cisplatin. Based on the plating efficiency, the surviving fraction was calculated by counting the number of colonies formed from adherent cells in the presence (13 μM for 4 h) or absence of cisplatin. **C** Cells were seeded at identical density and a scratch was introduced. Cell migration was assessed by measuring the closed distance after 24 h. Dots represent the mean of biological triplicate analyses per clone, lines represent mean ± SD of all clones. **D**–**F** Microscopy reveals pseudopodia-like structures (red arrows) in DTPs that are not present in naïve or parental OVCAR-3 cells. All pictures were taken with the same magnification; the bar represents 200 µm. *p < 0.05; **p < 0.01; ***p < 0.001; ****p < 0.0001.

We isolated isogenic treatment-naïve and DTP cells from the cisplatin sensitive HGSOC cell line OVCAR-3 [13]. The resulting 8 treatment-naïve (Naïve) and 12 DTP-derived cell clones were tested for viability, clonogenic survival and motility. Cell lines did not show significant differences in cell proliferation during passaging of cells. Both naïve and DTP-derived cells displayed similar viability following 48 h incubation in the presence of 13 µM cisplatin (Fig. 1A), with large inter-clonal heterogeneity. Treatment-naïve and DTP-derived clones also exhibited similar mean clonogenic survival 14 days after cisplatin treatment (Fig. 1B). In contrast, gap closure assays showed significantly enhanced motility and (Fig. 1C, Supplementary Data S1) pseudopodia-like structures in the DTP-derived clones, which were absent in naïve cells and in the parental OVCAR-3 cells (Fig. 1D–F).

### RNA sequencing distinguishes isogenic DTP-derived cells from naïve cells

**Fig. 2:**
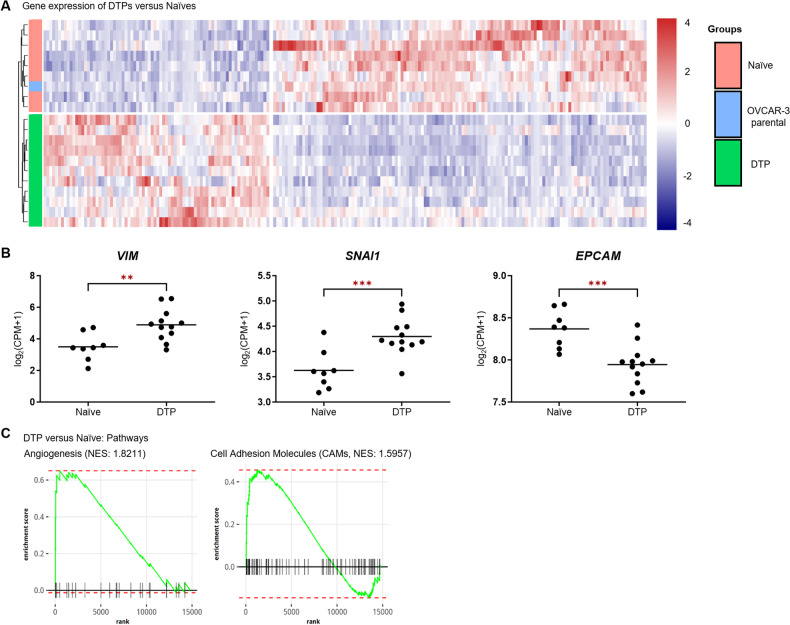
RNA sequencing distinguishes isogenic DTP-derived cells from naïve cells. **A** Heatmap of differentially expressed genes (DEG) between naïve and DTP cells compared to parental OVCAR-3 cells. **B** Gene expression of VIM, SNAI1 and EPCAM in each subclone of naïve versus DTP-derived cells was determined from the RNA-seq data. **C** Gene set enrichment analysis of gene expression comparing naïve and DTP clones. Genes of the signal pathways angiogenesis and cell adhesion molecules reveal differentially expressed genes that are upregulated in DTPs. *p < 0.05; **p < 0.01; ***p < 0.001; ****p < 0.0001.

Differential expression of parental, naïve, and DTP-derived cells was analyzed by RNA sequencing (RNA-seq) followed by clustering analysis. Although the overall gene expression patterns of naïve and DTP cells were very similar, with Pearson correlation ranging between 0.998–1.0 (Supplementary Data S2), the clustering analysis of the differentially expressed genes revealed that treatment-naïve clones closely resembled the parental OVCAR-3 cells but were distinguishable from DTP-derived clones (Fig. 2A). Significant differences between naïve and DTP clones were seen in the RNA expression of *VIM*, *SNAI1* and *EPCAM* (Fig. 2B). Gene set enrichment analysis (GSEA) of gene sets associated with hallmark gene signatures [14] (Fig. 2C) indicated that genes upregulated in DTP-derived cells are involved in angiogenesis and cell adhesion. The differential expression of *VIM*, *SNAI1* and *EPCAM* and the upregulation of genes related to angiogenesis and cell adhesion suggest a link to EMT.

### *ATF3* expression is elevated in aggressive DTP clones and abrogation of *ATF3* expression decreases their drug tolerance

**Fig. 3:**
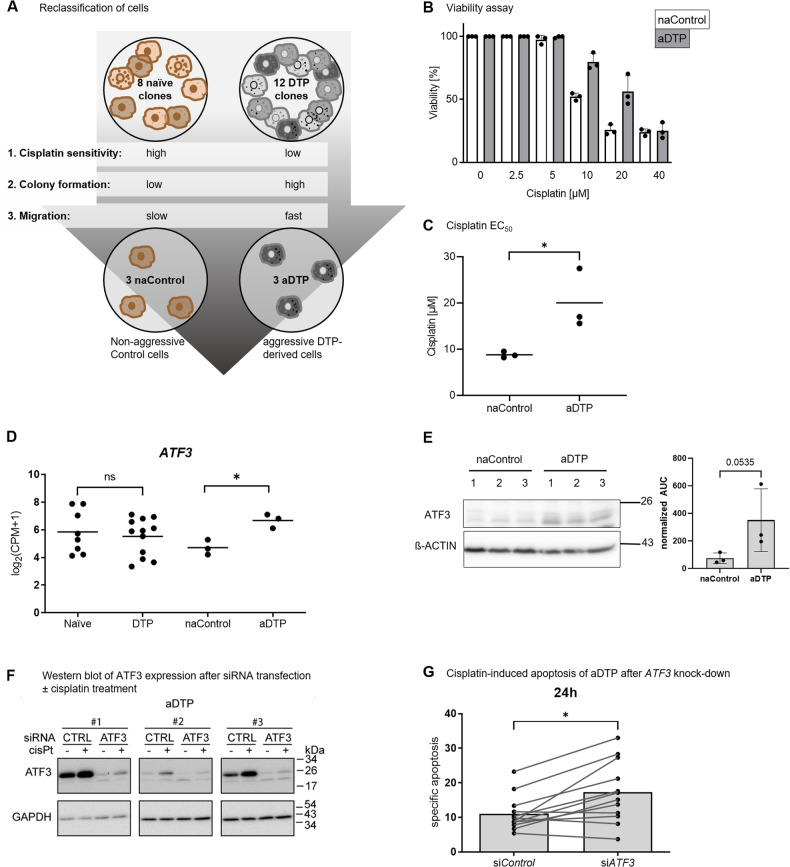
Aggressive DTP cells display increased *ATF3* expression, enhanced by cisplatin-treatment and providing a survival benefit. **A** Classification of aggressive drug-tolerant persister clones (aDTPs) and non-aggressive control clones (naControl) due to phenotypic and functional characteristics regarding cisplatin sensitivity, colony formation and migration. **B** aDTP and naControl cells were subjected to increasing cisplatin concentrations and cell viability was measured by CellTiter-Glo after 48 h of treatment. Values represent the average from all three clones in each group. **C** The half effective concentrations (EC50) of cisplatin for each of the 3 naControl and 3 aDTP clones were calculated from the viability assay data in (**B**). **D** *ATF3* mRNA expression in each clone within the indicated groups was determined from the RNA-seq data. **E** Western blot and densiometric analysis of ATF3 protein expression in parental OVCAR-3 cells and in naControl and aDTP clones. (Uncropped Western blots in Supplementary Data Original data). **F** Western blot analysis of ATF3 protein expression in the aDTP clones 1–3 in the presence or absence of 13 µM cisplatin for 24 h, after transient transfection with non-targeted (CTRL) or ATF3-targeted siRNA (ATF3). (Uncropped Western blots in Supplementary Data Original data). **G** Flow cytometric analysis of aDTP cells after transfection with control (siControl) or *ATF3*-targeted siRNA (si*ATF3*) in the presence of 13 µM cisplatin for 24 h. Cell viability was accessed by AnnexinV/PI staining. Data represents mean ± SD. *p < 0.05; **p < 0.01; ***p < 0.001.

As shown in Fig. 1A, B, there was substantial heterogeneity in the extent of cisplatin-induced cell death and clonogenic survival among individual clones within each group (naïve and DTP-derived). Conceivably, this phenotypic heterogeneity might have hindered the identification of additional transcriptional programs that distinguish DTP-derived cell populations. Of note, naïve clones that display increased survival under cisplatin treatment may be viewed as potential DTPs. We therefore compiled the most “aggressive” clones within the entire panel of DTP-derived and naïve clones, and denoted them aDTP (aggressive DTP) defined by the following attributes: (1) cisplatin-induced cell death below average, and (2) clonogenic survival above average, and (3) higher motility than average. As controls we selected the least aggressive clones, defined as those that not meeting any of the above three criteria (non-aggressive control, naControl). In total, three clones met the criteria for aDTP in the study, while three others met the criteria for naControl. Importantly, all three aDTPs defined in this manner were comprised within the panel of DTP-derived clones, while all three naControls were from the treatment-naïve set (Fig. 3A, Supplementary Data S3 and S4). The fourth non-sensitive clone was not selected as aDTP because it did not fulfill all three criteria for aDTP.

To further compare the drug response of naControl and aDTP cells, each clone was incubated with increasing concentrations of cisplatin (0–40 µM) and analyzed for cell viability. Remarkably, aDTP cells displayed higher viability than the naControl cells when exposed to 10 or 20 µM cisplatin (Fig. 3B), although 40 µM cisplatin resulted in efficient killing of both cell types. Overall, the EC50 of aDTP cells to cisplatin was significantly higher than that of naControl cells (Fig. 3C).

Remarkably, when comparing the RNA-seq data between the aDTP clones and the naControl ones, we noted that the stress response gene *ATF3* was expressed more abundantly in the aDTP cells and *ATF3* was not differentially expressed in naïve versus DTP-derived cells (Fig. 3D), indicating that this is an aDTP-specific feature. The aDTP clones displayed higher levels of ATF3 protein than their naControl counterparts (Fig. 3E). Accordingly, contingency analyses show a correlation of *ATF3* expression with cisplatin sensitivity and clonogenic survival but not with EMT characteristics (Supplementary Data S5). Moreover, the amount of ATF3 increased further when the aDTP cells were treated with cisplatin (Fig. 3F). Elevated ATF3 levels contribute to the increased resilience of the aDTP cells to cisplatin, as knock down of *ATF3* in the three aDTP clones (Fig. 3F) resulted in significantly greater cell death induction and partially restored the sensitivity of aDTPs to cisplatin (Fig. 3G). Hence, the upregulation of *ATF3* may be involved in the increased cisplatin tolerance of aDTP cells.

### aDTP-derived cells upregulate genes associated with stress and EMT-related pathways

**Fig. 4:**
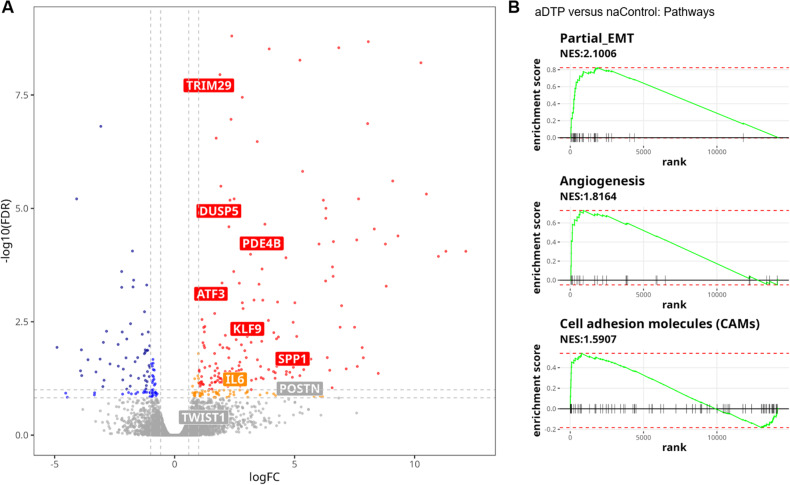
Aggressive DTP cells indicate upregulation in EMT and stress associated genes and pathways. **A** Volcanoplot of gene expression in naControl versus aDTP clones. *ATF3*-associated partial EMT gene signature genes are highlighted. Red/orange dots represent upregulated genes, red are determined by a cutoff (logFC > 1, together with *p*-value < 0.05), whereas orange by a cutoff (logFC < 1 but >0.59, meanwhile *p*-value < 0.1). Navy dots illustrate the genes satisfying a more stringent DGE threshold (logFC < −1 and *p*-value < 0.05), in addition blue dots on the leftside of volcanoplot show the genes with a flexible cutoff (logFC > −1 and < −0.59, meanwhile *p*-value < 0.1). **B** Gene set enrichment analysis of differentially expressed genes upregulated in aDTP versus naControl clones.

Next, we compared the overall gene expression based on RNAseq data of aDTP and naControl cells. The volcano plot in Fig. 4A showed all differentially expressed genes of aDTP and naControl cells. We identified a subset of EMT- and stress-associated genes that were significantly either upregulated in aDTP cells or at least showed a trend towards upregulation highlighted in the volcano plot. Conversely, the epithelial marker EPCAM was significantly downregulated in aDTP cells, relative to the respective control (data not shown). Comparison of the aDTP and naControl clones identified a group of 32 genes and this group was named “*ATF3*-associated partial EMT signature” (short: “partial EMT”, Supplementary Data S6 and S7). The gene set origins from the MsigDB hallmark EMT gene set and genes based on differential gene expression from in vitro RNA-seq results were added to the gene set.

The “partial EMT” gene set was also significantly enriched in the comparison between the DTP-derived vs naïve clones described earlier (Supplementary Data S8), but with a normalized enrichment score (NES) of 1.8, which is lower than the NES of 2.1 in the naControl versus aDTP comparison (Fig. 4B). The hallmark gene signatures “angiogenesis” and “cell adhesion molecules” were also upregulated in the aDTP clones. The fact that “angiogenesis” and “cell adhesion molecules” were also strongly enriched in the comparison between DTP-derived and naïve clones (Fig. 2C) suggests that upregulation of those pathways is not specific to aggressive DTP cells, but rather is a general feature of all DTP-derived clones.

### Expression of EMT and stress response genes is associated with clonal expansion in an in vivo relapse model

**Fig. 5:**
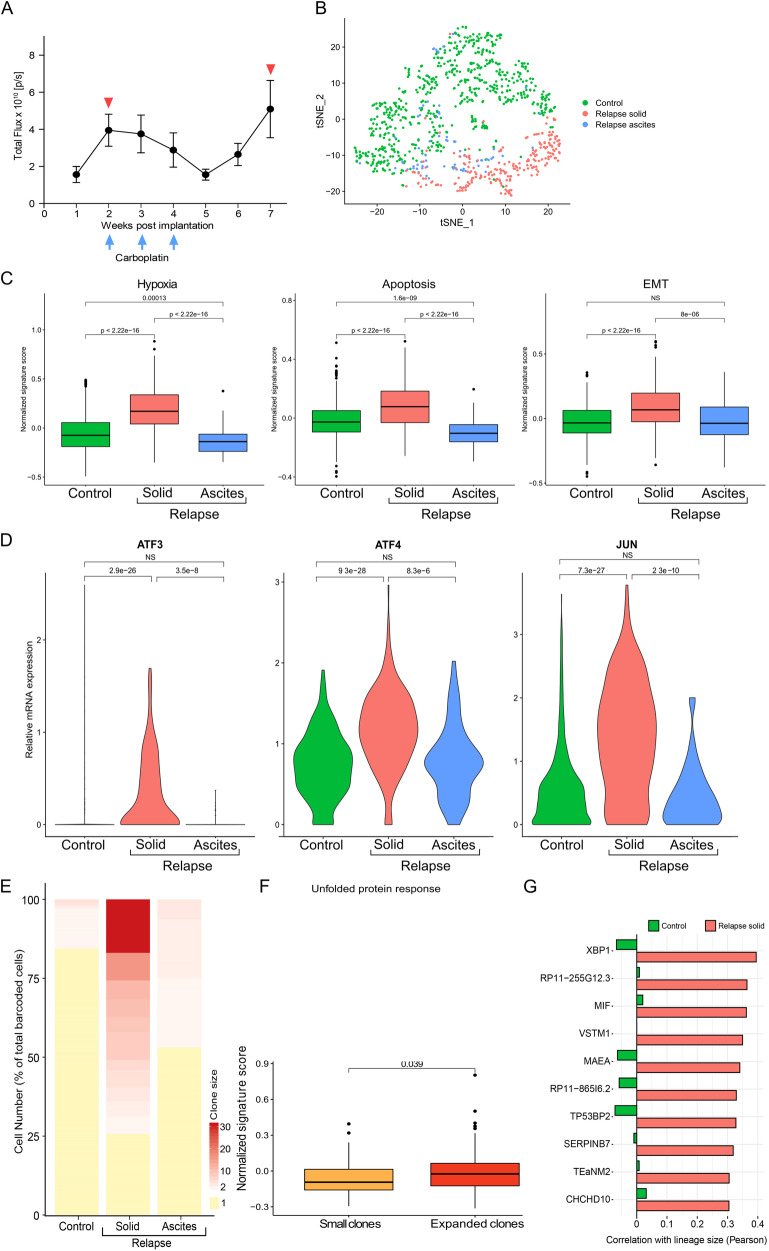
ScRNA-seq of HGSOC xenografts reveals upregulation of EMT and ER stress genes during relapse*.* **A** OVCAR-8 Watermelon (WM) cells expressing luciferase were injected intraperitoneally and mice were imaged over a time course of two months. Images were taken weekly and total flux was calculated for each time point to determine tumor growth. Blue and red arrows indicate carboplatin administration and sample collection for single cell RNA profiling, respectively. **B** t-SNE plot of OVCAR-8-WM cells harvested from mice. 930 single cells from control and relapsed samples were profiled by single-cell RNA-seq. Cells are colored by sample. **C** Solid relapse samples showed upregulation of Hallmark pathway signature genes associated with hypoxia, apoptosis and EMT. **D** The ER stress marker genes *ATF3*, *ATF4* and *JUN* are significantly upregulated in the solid relapse samples. **E** Lineage analysis of clonal expansion in relapsed samples indicated an increase in clone size in both solid and ascites samples. **F** Hallmark pathway signature genes of the unfolded protein response (UPR) are significantly upregulated in the most abundant lineages. **G** Correlation of gene expression with clone size shows a strong correlation of solid relapse with the ER stress response gene *XBP1* but no correlation with clone size among untreated cells.

Next, we set out to identify genes that are associated with drug tolerance in vivo. To this end, we utilized a cell line derived xenograft (CDX) model of HGSOC (OVCAR-8 cells) expressing the both a luciferase reporter and the Watermelon vector (WM) [15]. The WM vector library is an expressed lentiviral barcode library that enables simultaneous tracing of the lineage as well as the transcriptional state of cells. Specifically, WM-OVCAR-8 cells were generated (see methods), expanded and injected into the peritoneal cavity of immunocompromised NRG mice. Two weeks post-implantation, mice were administered carboplatin once a week for three consecutive weeks, and tumor burden was monitored via bioluminescent imaging (Fig. 5A). Subsequently, solid tumors and ascites samples were collected, dissociated and subjected to single cell RNA sequencing (scRNA-seq).

t-SNE analysis of more than 930 tumor cells isolated from solid metastases, ascites and treatment-naïve mice, revealed a clear separation between solid and treatment-naïve samples, with ascites cells falling within both clusters (Fig. 5B). GSEA of hallmark pathways of all differentially expressed genes showed enrichment for gene signatures involved in hypoxia, apoptosis and EMT in the solid tumor cells (Fig. 5C). Importantly, expression of multiple stress markers, including *ATF3*, *ATF4*, and the *ATF3* binding partner *JUN*, showed a significant upregulation of these markers in the relapsed solid metastasis fraction (Fig. 5D). To further study clonal dynamics, we assigned each cell to a clone based on the expressed lineage barcode transcript. Analysis of barcode frequency revealed an increase in clone size in both solid and ascites samples following treatment (Fig. 5E). Nevertheless, greater clonal expansion was observed in the solid tumor, where 75% of lineages were comprised of more than one cell following chemotherapy.

To uncover what drives clonal expansion in the solid relapsed tumor, we looked for pathways that were differentially expressed in expanded clones, versus clones comprised of only one cell. In line with our in vitro findings, the stress signature was significantly upregulated in expanded clones following treatment (Fig. 5F). Interestingly, the most abundant solid relapse lineage revealed a significant upregulation of the ER stress response gene *XBP1* (Fig. 5G), which was also markedly correlated with overall solid relapse clone size (Pearson correlation of 0.4, Fig. 5G). These findings support the role of the ER stress response in promoting DTP fitness in vivo.

### Single-cell RNA-seq analysis of HGSOC patient cells identifies five subpopulations of EPCAM+ cells and reveals a subgroup that closely mimics DTP

**Fig. 6:**
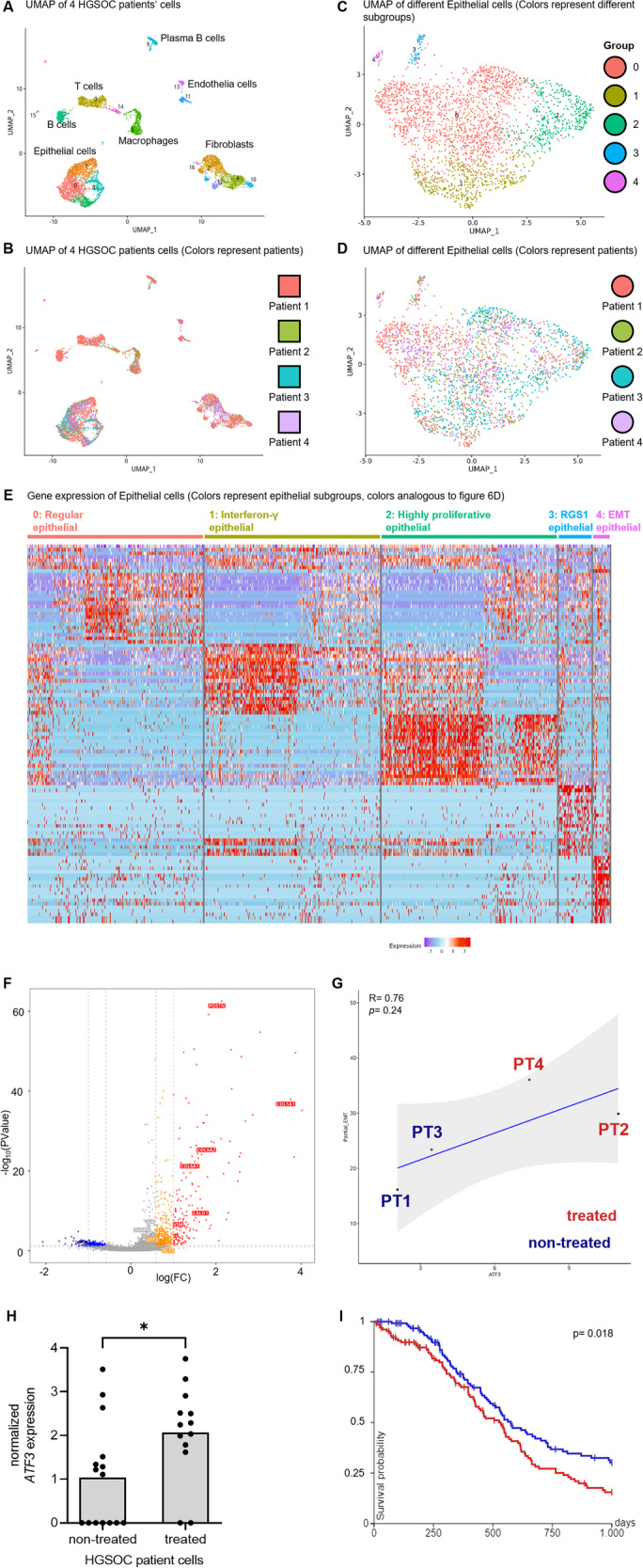
Single-cell RNA-seq of HGSOC patient cells identifies five distinct *EPCAM* + cell subpopulations and reveals a subgroup that closely mimics DTP. **A** UMAP of scRNA-seq data from 4 HGSOC patient samples colored by cell type and subtypes. **B** UMAP of scRNA-seq data from 4 HGSOC patient samples colored by patients. **C** UMAP of *EPCAM* + HGSOC cells clustered without external specifications and colored by epithelial subgroups. **D** UMAP of *EPCAM* + HGSOC cells colored by patients. **E** Heatmap showing the clustering result of HGSOC epithelial cells and expression levels of marker genes. Each column represents an epithelial subtype and rows represent marker genes. Colors indicate the expression levels as shown in the scale bar. (Epithelial subgroup colors are analogous to Fig. 6D). **F** Volcano plot shows all differentially expressed genes between epithelial subgroup 4 and groups 0–3. Highlighted genes belong to the partial EMT gene signature. Red/orange dots represent upregulated genes, red are determined by a cutoff (logFC > 1, together with *p*-value < 0.05), whereas orange by a cutoff (logFC < 1 but >0.59, meanwhile *p*-value < 0.1). Navy dots illustrate the genes satisfying a more stringent DGE threshold (logFC < −1 and *p*-value < 0.05), in addition blue dots on the leftside of volcanoplot show the genes with a flexible cutoff (logFC > −1 and < −0.59, meanwhile *p*-value < 0.1). **G** Correlation of *ATF3* gene expression and partial EMT signature of epithelial cells from 4 HGSOC patients. The pearson correlation analysis indicates a linear correlation between *ATF3* gene expression and expression of partial EMT gene signature among the four HGSOC patients. **H** Basal *ATF3* gene expression in cells of group 4 (*n* = 28), based on primary HGSOC RNA-seq data. **I** Kaplan-Meyer progression-free survival plot from TCGA HGSOC data (259 patients). Blue and red indicate patients with low and high *ATF3*-associated partial EMT gene expression, respectively.

To examine whether the aDTP subpopulation is present in patients, we re-analyzed published scRNA-seq data of four late stage HGSOC patients (patient (PT) 1 and PT3 treatment-naïve, PT2 and PT4 with neoadjuvant therapy, International Federation of Gynecology and Obstetrics (FIGO) IIIC) [16]. Patient cell types were hierarchically clustered and visualized in Uniform Manifold Approximation and Projection (UMAP) plots. The results show that the individual clusters were formed according to cell type (Fig. 6A) and were not organized in a patient-specifically (Fig. 6B). When restricting the analysis on *EPCAM*+ cells using the default settings without external filtering it revealed five distinct epithelial subsets (Fig. 6C); clusters were again not patient-specific (Fig. 6D). These epithelial subtypes were assigned with respect to the upregulated genes in each subtype, based on molecular expression patterns. The most distinctive biological feature of each group, based on its top regulated genes, is implied in the group name (Fig. 6E): “regular epithelial” (group 0), “Interferon-γ epithelial” (group 1), “Highly proliferative epithelial” (group 2), “RGS1 epithelial” (group 3) and “EMT epithelial” (group 4). A volcano plot labeled for partial EMT genes confirmed that the majority of genes in the analogously named group are upregulated, including *ATF3*, which is one of the significantly enriched genes (Fig. 6F).

Eventually, the cell numbers of *EPCAM*+ patient cells and the fraction of the five epithelial subgroups were determined for each patient (Table 1). Between 0.4–1.8% of epithelial cells in each patient were assigned to the “EMT epithelial” subgroup, in line, with the frequency of DTP cell occurrence reported in literature [3]. Interestingly, the neoadjuvant patients displayed 1.3% and 1.8% EMT-like epithelial cells while the non-treated displayed 0.4% and 1.2% EMT-like cells. The significance of this observation has to be confirmed in larger patient cohorts.

**Table 1:** Epithelial subtypes derived from the scRNA-seq data of four HGSOC patient.

#	Group names	PT1 (Σ 982 cells)	PT2 (Σ 312 cells)	PT3 (Σ 817 cells)	PT4 (Σ 493 cells)
		Non-treated	Neoadjuvant therapy	Non-treated	Neoadjuvant therapy
0	regular epithelials	701 (71.4%)	222 (71.2%)	296 (36.2%)	410 (83.2%)
1	interferon-γ epithelials	140 (14.3%)	42 (13.5%)	259 (31.7%)	27 (5.5%)
2	highly proliferative epithelials	100 (10.2%)	32 (10.3%)	249 (30.5%)	42 (8.5%)
3	RGS1 epithelials	29 (3.0%)	12 (3.8%)	10 (1.2%)	5 (1.0%)
4	EMT epithelials	12 (1.2%)	4 (1.3%)	3 (0.4%)	9 (1.8%)

Absolute and relative cell numbers of HGSOC patient cells were analyzed with respect to epithelial subgroups.

To assess the correlation between *ATF3* gene expression and the *ATF3*-associated partial EMT gene signature of all patient cells of group 4, cells from previously treated patients were compared with treatment-naïve patients. The quantitative values of *ATF3* gene expression suggest a correlation with partial EMT gene expression of the four patients, (Fig. 6G, *p* > 0.05, due to *n* = 4). A significant correlation between *ATF3* and partial EMT is seen when the individual epithelial subgroups of the 4 patients are analyzed (Pearson correlation: R = 0.54, *p* = 0.014, Supplementary Data S9). Furthermore, Fig. S9 shows that the epithelial cells of the previously treated patients are characterized by higher *ATF3* and partial EMT expression than the non-treated ones. Of all four patients, the EMT epithelial subgroups cluster spatially close to the upper part of the correlation plot.

Finally, the *ATF3* expression of all cells from group EMT epithelial was analyzed (Fig. 6H) and we found a heterogeneous *ATF3* gene expression, closely resembling the heterogeneity of in vitro DTP cells. Significantly higher *ATF3* expression was observed in EMT epithelial cells from previously treated patients compared to non-treated (*p* = 0.026). This may indicate cells with a higher *ATF3* gene expression have survived therapy and might have a more aggressive phenotype. Consequently, we hypothesized that the “EMT epithelial” group might include DTP cells occurring in patients. This EMT/*ATF3* high subpopulation was found in each patient (Supplementary Data S10–13).

In order to ask whether *ATF3* gene expression relate to a more aggressive clinical course, TCGA data from 259 HGSOC patients were obtained from University of California Santa Cruz (UCSC) Xena Browser [17]. Patient samples were divided into high (130 patients) and low (129 patients) expression with respect to their gene expression of the gene list *ATF3*-associated partial EMT (S7). The result showed a significantly higher progression-free survival of the group with lower *ATF3*-associated partial EMT gene expression (Fig. 6I).

## Discussion

The in vitro and in vivo results presented here suggest that expression of EMT and stress response genes, particularly *ATF3*, might be involved in the emergence of aggressive DTP subpopulations. The expression of EMT-associated genes appears to be a feature of DTP cells and *ATF3* expression is associated with an aggressive biological behavior and reduced cisplatin-induced cell death of these cell clones in vitro. This subpopulation was also found in a relapse mouse model and in HGSOC patient cells. This population of HGSOC cells may be involved in the tumor recurrence characteristic for late stages of HGSOC.

DTP-derived cells generated by pulse-treatment of cisplatin, showed enhanced migratory capacity and a spindle-shaped morphology. A similar phenotype was described in HGSOC to be associated with aggressive disease and impaired drug response [9, 18]. This phenotype of DTP cells reflects features of the epithelial-mesenchymal transition (EMT), which is also associated with metastasis formation [5].

RNA-seq data analyses of our HGSOC models revealed upregulation of EMT-associated gene set signatures in DTP and aDTP cells in vitro, in the CDX model and in vivo. We found increased EMT markers such as *VIM* and *SNAI1*, and downregulated epithelial marker *EPCAM* indicating a partial EMT program. EMT and its counterpart mesenchymal-to-epithelial transition (MET) are not all-or-none processes [19], but rather define a spectrum, ranging from purely epithelial to purely mesenchymal via intermediate phenotypes. These intermediate states, known as partial, incomplete or intermediate EMT, [20–24] have been shown to be required for metastasis formation in a mouse cancer model [25]. Thus, EMT as a dynamic process not requiring genetic mutations is an attractive mechanism underlying persistence of tumor cells upon drug treatment [26].

A large functional heterogeneity was observed in naïve and DTP cells generated from a cell line. In *TP53* mutated cells, clonal diversity is expected, owing to genome instability [27, 28]. Accordingly, this clonal heterogeneity was similar in control and DTP cells. In contrast, aDTP clones consistently differed from naControl cells by an increased ATF3 RNA and protein expression and contribute to protect against cisplatin-induced cell death in aDTP cells.

The role of ATF3 seems to be context dependent. *ATF3* has been implicated in tumor promotion, metastasis, and reduced patient survival. In breast cancer, *ATF3* contributes to pro-metastatic changes in the tumor microenvironment that are enhanced by chemotherapy [29]. In a prostate cancer model, ATF3 functions to promote metastasis [30]. Cisplatin-induced *ATF3* expression in a MAPK pathway-dependent manner has been described in various cell lines and mouse models [31]. Increased *ATF3* gene expression in 31 HGSOC patient samples and in cell lines was observed after neoadjuvant chemotherapy and significantly increased in platinum resistant cells [32]. Not so many ATF3 downstream targets in the context of cancer are known. In skin cancer tissue, ATF3 accumulates and promotes the proliferation of skin cancer cells by inhibiting p53 expression and then activating Stat3 phosphorylation [33]. ATF3 promotes colon cancer metastasis [34] and is described to build a stable complex with Smad4 that could be responsible for the activation of genes that participate in TGF-β1-mediated breast cancer progression [35]. Furthermore, ATF3 and ATF4 form dimers to stimulate MMP13 expression after stimulation with TGF-b11 [35] and CHAC1 (cation transport regulator-like protein 1) is described as downstream target of ATF3 in human aortic endothelial cells [36].

To identify aDTPs in an in vivo setting, HGSOC cells labeled with the watermelon library [15] were injected into NRG mice. After platinum-based therapy, scRNA-seq revealed enriched gene signatures of the hypoxia, apoptosis, and EMT signaling pathways in solid tumor metastases. Hypoxia is an important driver of angiogenesis in terms of oxygen and metabolite delivery [37]. Increased hypoxia often induces angiogenesis via the hypoxia inducible factor-1 (HIF-1) transcription factor, which has multiple angiogenesis modulating target genes [38]. As previously described, an important link exists between angiogenesis and EMT, which may also be promoted by increased hypoxia, which was shown to trigger EMT in breast, prostate and oral cancer [39]. This is in line with our findings that the gene signatures of the EMT signaling pathways are increased in solid relapse cells.

Interestingly, “apoptosis” was also enriched in the solid relapse cells. Approximately 50% of cells come from expanded lineages consisting of >10 cells (Fig. 5E). It is conceivable that these expanded lineages have a specific anti-apoptosis pattern in contrast to the non-expanded lineages in the control and ascites group. Increased anti-apoptotic gene expression could give these cells a survival advantage. For example, NOTCH1 overexpression has been shown to enhance cell growth, migration and invasion and simultaneously upregulate anti-apoptotic BCL-2 in salivary adenoid cystic carcinoma [40]. This interesting possibility requires further investigation in our model. At the single gene level, persister cells showed increased expression of *ATF3*, *ATF4*, and *JUN*, suggesting an increased stress response. The most abundant cell lineage after treatment showed increased *XBP1* expression and overall increased expression of members of the ER stress response pathway. No correlation between *XBP1* expression and clone size was observed before treatment, suggesting that upregulation of *XBP1* is specific to cells that survive drug treatment and have evolved to metastasis. A stress-induced transcriptional profile, including *ATF3* and *Jun* enrichment was described during platinum-based chemotherapy in HGSOC, and high expression of this transcriptional profile was associated with poor progression-free survival [41]. Interestingly, unlike UPR, the EMT signature and *ATF3* are not associated in our in vivo model with expended solid clone size (adjusted *P* value > 0.05). We therefore propose that increased expression of stress response genes may be a feature of HGSOC transition to aDTP.

The single cell RNA-seq data of epithelial cells of HGSOC patients clustered cells in 5 subgroups. Olalekan et al. have identified similar subsets in a pan-cell analysis including a minor fraction of EMT positive cells [16]. When using the gene list defined in aDTP cells in vitro to analyze patient data, we found cells resembling the profile of DTP cells. This expression pattern was present in all four patients studied. Others have also found an EMT subset among secretory epithelial cells [42] or cell clusters with high EMT gene expression in a minority of cancer cells [43, 44] correlating with a poor prognosis for HGSOC patients [45]. Moreover, the stress-associated transcriptional profile was found to be enriched after chemotherapy, indicating that these cells possess a survival advantage during therapy [46]. The relative abundance of DTP cells that we observed in samples from HGSOC patients is in line with what has been described in the literature ranging from 0.3–5% [3]. Cells with very high and very low *ATF3* expression were also identified in this subgroup, analogous to our in vitro results. In contrast, low expression of *ATF3* has been reported in ovarian cancer cells compared to non-cancerous ovarian epithelial cells [47]. This discrepancy might be explained that increased *ATF3* expression is observed only in a subgroup of tumor cells comprising only a few percent of all cells in the tumor and therefore this subgroup could not be identified in bulk sequencing analyses.

We found an increased partial EMT and stress-associated expression pattern in proliferating and aggressive DTP cells in each model examined, respectively. Increased expression of *ATF3* and partial EMT might be markers of the development of aggressive DTP cells and cells expressing this phenotype are important for recurrence and metastasis in HGSOC patients.

## Materials and methods

### Cell culture

OVCAR-3 cells were purchased from ATCC (Rockville, MD, USA) in 2007 and cultured in RPMI-1640 (Biochrom, Germany) supplemented with 10% fetal bovine serum (FBS, Sigma-Aldrich, Germany), 1% penicillin/streptomycin (Thermo Fisher Scientific) and 2 mM L-glutamine (Biochrom) up to 25 passages. OVCAR-8 cells (obtained from the lab of Joan Brugge/Harvard Medical School in 2017) were cultured in 1:1 MCDB 105 medium (Sigma-Aldrich) and Medium 199 Earle’s Eagles medium (Thermo Fisher Scientific) supplemented with 10% fetal bovine serum (Gibco) and 1% penicillin/streptomycin (Sigma-Aldrich) up to 7 passages. Cell lines were regularly tested for mycoplasm and authenticated by STR profile analysis.

### Watermelon cell line construction

OVCAR-8 cells were transduced with pLX302 Luciferase-V5 puro lentiviral vector (Addgene; plasmid #47553) and selected with 2 µg/mL puromycin. The OVCAR-8-Luc cells were transduced using the Watermelon lentivirus by spin infection using 8 μg/mL polybrene at 2500 rpm for 30 mins at 30 °C. After 24 h incubation with virus, the medium was changed, and 72 h post-infection the cells were sorted for mNeonGreen expression. To ensure that the majority of cells were labeled with a single barcode per cell, for watermelon lentiviral infection, a target multiplicity of infection (MOI) of <0.3 was used, corresponding to less than 30% mNeonGreen expressing cells 72 h post-infection. Sorted cell populations, 5 × 10^4^ each, were expanded in culture for three passages, aliquoted to 1 × 10^6^ cells per vial and stored in liquid nitrogen.

### Mouse experiments

Animals were maintained in compliance with the guidelines and protocols approved by the Animal Care and Use Committees at the Massachusetts Institute of Technology, USA (approval no. 1017-097-20). Briefly, 2 × 10^6^ luciferase expressing watermelon-library barcoded OVCAR-8 cells were resuspended in 100 μL of sterile PBS. Cells were injected into the intraperitoneal cavity of 10, 6–8-week-old, female, NRG (NOD-*Rag1*^null^ *IL2rg*^null^, NOD rag gamma), immunocompromised mice (purchased from The Jackson Laboratory, USA). Mice were then randomly assigned into 2 groups at 5 mice per group. One group was left untreated (i.e. treatment-naïve) while the other group of mice (~25 g) were treated with 70 mg/kg of clinical-grade carboplatin (Hospira Inc., obtained from the Dana Farber Cancer Institute Pharmacy, USA) two weeks post-implantation, via intraperitoneal injections. Drug treatments were administered once per week, for 3 consecutive weeks. Tumor growth was monitored via bioluminescence in live animals using the IVIS Spectrum in vivo imaging system. Bioluminescent images were analyzed using Living Image software (PerkinElmer, ORT). Investigators were not blinded to the group allocation during the experiment. Treatment-naïve mice were euthanized at the time of the first carboplatin treatment (*n* = 5 mice). Carboplatin-treated animals were sacrificed 7 weeks post-injection (*n* = 4 mice; 1 mice succumbed to tumor burden prior to 7 week endpoint and was therefore not included in the final tumor assessment). Intraperitoneal washes with PBS were performed to collect residual tumor cells located in the intraperitoneal cavity. Collected wash samples from each mouse were pooled and spun at 1200 rpm for 5 mins. Cell pellets were incubated with red blood cell lysis buffer (BioLegend) for 5 mins, washed with PBS and incubated with 0.15% trypsin solution at room temperature for 10 mins, with gentle pipetting every 5 mins. Trypsinization was quenched with culture medium. Cells were pelleted by centrifugation, washed with PBS, and then resuspended in 0.04% BSA/PBS at a concentration of ~3000 cells/μL. Cells were passed through a 40 μm cell strainer prior to single cell RNA-sequencing. Using as control, the cells that were harvested before treatment, in the common practice in lineage-based studies (For recent examples please see: Harmange Nature Comm 2023 [48] and Goyal, Nature 2023 [49].

Pooled omental metastases were minced with a razor blade and digested in RPMI containing 2 mg/mL collagenase (Sigma–Aldrich) and 100 U/mL hyaluronidase (Sigma–Aldrich) in a rotator at 37 °C for 1 h. To derive single-cell suspensions, dissociated cells were washed twice in PBS and filtered through a 70 and 40 μm cell strainer. Cells were resuspended in 0.04% BSA/PBS at a concentration of ~3000 cells/μL prior to single cell RNA-sequencing.

### Generation of naïve and DTP cell clones

To generate OVCAR-3 control clones 10^3^ cells were seeded in petri dish (Ø10 cm) and individual clones were isolated and expanded. OVCAR-3 cells (1.2 × 10^6^) were incubated (13 µM cisplatin, 4 h) and after recovery the cells were again incubated cisplatin (13 µM cisplatin, 4 h). Subsequently, single DTP clones were isolated and expanded. The clones were cryoconserved for further use.

### Cell viability assay

- Apoptosis detection
  One day prior to experiments, 10^5^ cells per well were seeded in a 6-well plate or 3 × 10^5^ cells were seeded per T-25 cell culture flask. Cells were incubated with 13 µM cisplatin (Teva GmbH, Ulm, Germany) for 48 h, further collected and washed in Annexin binding buffer (ABB: 140 mM NaCl, 2.5 mM CaCl_2_, 10 mM Hepes, pH 7.4). Cells were stained for 10 mins with 1:20 Annexin V-APC (ImmunoTools, Friesoythe, Germany) and 1.25 µg/mL propidium iodide in 300 µL ABB. Flow cytometric analysis was performed using a FACS Lyric flow cytometer and the software BD FACSuite V1.2.1 (Becton Dickinson GmbH, Heidelberg, Germany).
- Cell Titer Glo assay
  In 80 µL cell culture medium, 4000 cells per well were seeded in 96-well plate. On the next day, 13 µM cisplatin was added for 48 h and 95 µL CTG reagent (Promega, Walldorf, Germany) was added. Samples were measured with the 2300 EnSpire Multimode Plate Reader (Perkin Elmer).

### Colony formation assay

To calculate the plating efficiency (PE), 500 cells were seeded per T-25 flask and colonies were counted after 7 days. Plating efficiency was calculated as follows: PE = colonies count/seeded cells × 100%. For the surviving fraction (SF) 2 × 10^5^ cells were seeded per T-25 flask and after 24 h the cells were incubated with 13 µM cisplatin for 4 h. After 1–2 weeks, the colonies were counted, and the SF was calculated by: SF = colonies count/seeded cells xPE. To count the colonies, the cells were fixed with cold 70% ethanol for 5 mins and stained with Mayer’s hematoxylin solution (Sigma–Aldrich).

### Gap closure assay

A total of 1.7 × 10^5^ cells were seeded per 12-well plate well in triplicates. On the next day, a scratch was introduced with a 200 µL pipette tip and cells were incubated with 30 µM Mitomycin C to prevent cell proliferation. Pictures were taken directly after the scratch and after 24 h. The gap distance was measured with ImageJ.

### Antibodies

Antibodies used were: anti-ATF3 (Santa Cruz, #sc-188), anti-GAPDH (Cell Signaling, #2118), anti-β-ACTIN (Merck, #A5541). Secondary anti-mouse (#7076 S) and anti-rabbit (#7074 S) horseradish peroxidasecoupled antibodies were from Cell Signaling.

### Western blot

Protein expression was analyzed by Western blot as described elsewhere [50]. Equal amounts of protein (typically 30–40 μg) were separated by SDS-PAGE and blotted (Biometra FastblotTM, Analytic Jena) onto nitrocellulose membrane (0.1 μm; GE Healthcare) by semi-dry blotting (1 mA/cm^2^, 1 h). Quantitative densiometric analyses from Western blots were conducted with imageJ.

### RNA isolation

RNA was isolated with the RNeasy Mini KIT (Qiagen, Hilden, Germany) according to the manufacturer’s protocol with adaptions: For lysis, 0.14 M β-Mercaptoethanol was added to RLT buffer and final elution was performed with 40 µL nuclease-free water.

### Reverse transcriptase quantitative Real-Time qPCR

For TaqMan qPCR cells were harvested, washed twice with cold PBS and RNA isolated (see RNA isolation). TaqMan assays (Applied Biosystems, Foster City, CA, USA) were performed with the 7500 real-time PCR system (7500 software V2.3).

### siRNA knockdown

The cells were transfected with Smart-Pool siRNA according to the manufacturer´s protocol (Horizon Discovery, Waterbeach, UK, 5 µL Dharmafect-1 + 1 µL non-targeted control- or *ATF3*-siRNA final concentration 50 µM in 400 µL OptiMEM). Subsequently, the medium was replaced with fresh medium after 6 h. The next day, cells were incubated with 13 µM cisplatin for another 24 h, and were harvested for flow cytometric (Annexin V) and gene expression analyses (qRT-PCR).

### Statistical analysis

Cell culture experiments were analyzed from *n* = 3 biological replicates, the pragmatic sample size number commonly used for this type of data. Data are expressed as mean ± SD. For statistical analyses, Student’s *t*-tests were performed with Prism (GraphPad, 5.04). The confidence level of 95% was chosen and *P* < 0.05 or *p*_adj_ < 0.05 was considered as significant, respectively.

### OVCAR-3 RNA sequencing and data analysis

The isolated RNA was prepared with a stranded mRNA-seq library and sequencing was done with 2 × 75 bp paired-end reads (Illumina, San Diego, USA). FASTQ read files were obtained, adapters were removed with trim_galore, and low-quality read ends were clipped. The filtered sequence reads were mapped to reference genome using STAR aligner, the aligned gene count data were generated with featureCounts.

### Watermelon-OVCAR-8 cell analysis

#### Single cell RNA sequencing

Following dissociation, cells were spun down and approximately 9000 single cells per sample were loaded to the Chromium Controller.

### Data analysis

Reads were mapped to the GRCh38 human transcriptome using cell ranger 2.1.0, and transcript-per-million (TPM) was calculated for each gene in each filtered cell barcodes sample. TPM values were then divided by 10, since the complexity of single-cell libraries is estimated to be on the order of 100,000 transcripts. For each cell, we quantified the number of genes expressed and the proportion of the transcript counts derived from mitochondrial encoded genes. Cells with either <2000 or more than 7200 detected genes or >0.1 mitochondrial fraction were excluded from further analysis. The resulting expression matrix was filtered to remove genes detected in <3 cells. All the above steps were done using the Seurat R package (version 3.0). Next, we used Seurat to perform differential expression analysis using the following thresholds: adjusted *p* value lower than 0.001 and a |log2FC | >0.2. MSigDB [14] was then used to perform an enrichment analysis on differentially expressed genes. Last, we calculated signature score per cell [51] and the mean signature score per sample for the differentially expressed gene signatures.

### Mapping of cell lineage

To increase lineage detection rate, lineage barcode PCR dial-outs were used for generating a cell-barcode (CB) lineage-barcode (LB) map as previously described [15].

### HGSOC patient material single cell RNA-seq and data analysis

The raw data for scRNA-seq were extracted from the publication of Olalekan S et al. in 2021 and were obtained from NCBI GEO (accession number: GSE147082). We selected the scRNA-seq data from 4 patients PT2 (GSM4416538, =PT1), PT3 (GSM4416536, =PT2), PT4 (GSM4416534, =PT3) and PT5 (GSM4416537, =PT4). Other patient data were not HGSOC samples. Data were integrated and investigated using Seurat 4, where the batch effect was carefully removed. Cells with either <500 or >5000 detected gene features, or >0.25 mitochondrial fraction were excluded from further analysis. Meanwhile the resulting expression matrix was filtered to remove genes detected <10 cells. MSigDB (ver. 7) was used to perform a gene set enrichment analysis when comparing different cell clusters. Cell types was identified and annotated with the help of SingleR package (ver. 1.8, [52]). Volcano plots and bar plots were generated using ggplot2.

## Conclusion

Our results propose that increased *ATF3* expression together with partial EMT promotes the development of aggressive DTP in vitro and in vivo. Concordantly, we found the same signatures in a subset of patient cells suggesting that these cells may be associated with metastatic disease in HGSOC patients.

### Supplementary information

Supplementary data

Original Data Western blots

## Data Availability

The datasets used and/or analyzed during the current study are available from the corresponding authors on reasonable request.
