# Supplementary material for: ATF3 characterizes aggressive drug-tolerant persister cells in HGSOC
Source: Cell Death Dis. 2024 Apr 24;15(4):290. doi: 10.1038/s41419-024-06674-x (PMC11043376; doi:10.1038/s41419-024-06674-x)
Supplement: Supplementary file 1 — Supplementary data [file 41419_2024_6674_MOESM1_ESM.pdf]

Supplementary data of manuscript: “***ATF3* characterizes aggressive drug-tolerant persister cells in HGSOC**”

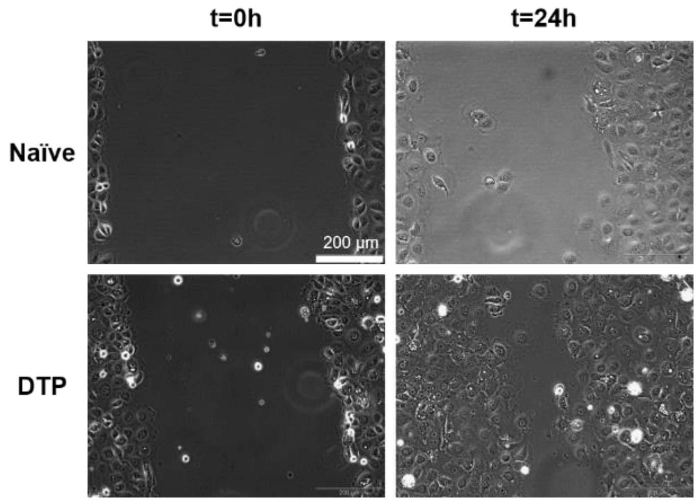

S1 Representative images of scratch assays. Data is shown in Figure 1C.

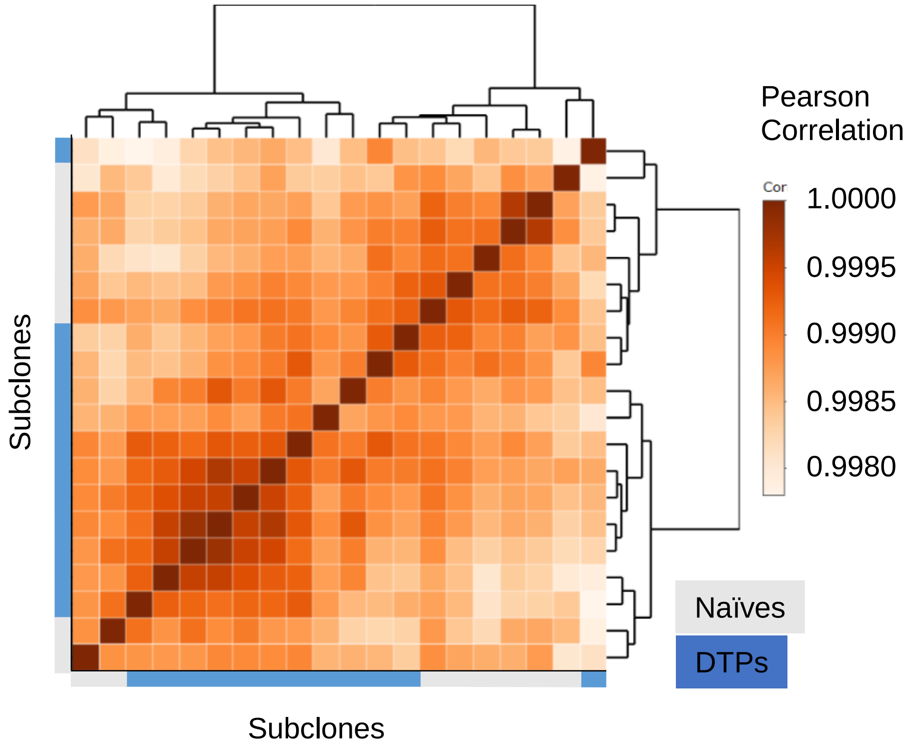

S2 Pearson correlation distance comparing the overall gene expression of naïve and DTP clones.

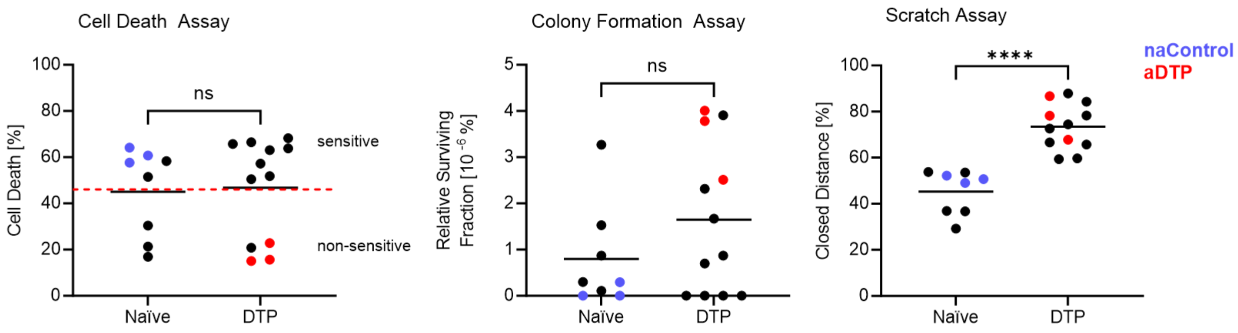

S3 Color label of naControl (blue) and aDTP (red) clones of the data in Figures 1A-C.

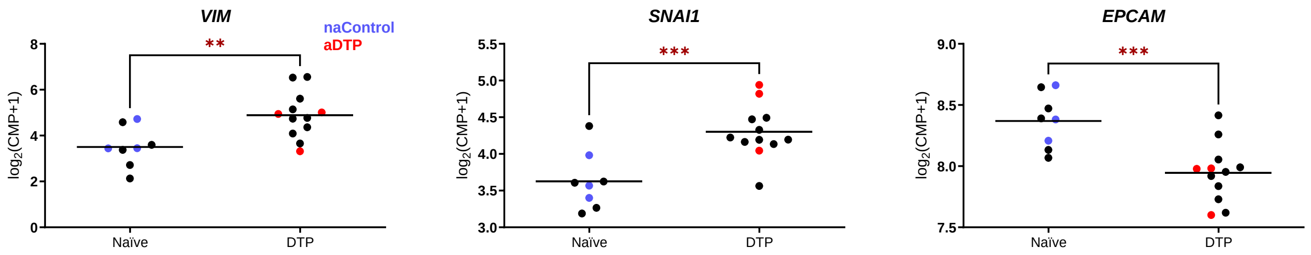

S4 Color label of naControl (blue) and aDTP (red) clones of the data in Figures 2B.

A

	Death high	Death low
ATF3 high	5	6
ATF3 low	8	1

p = 0.058

B

	Colonies high	Colonies low/no
ATF3 high	7	4
ATF3 low	1	8

p = 0.025

C

	Migration high	Migration low
ATF3 high	5	6
ATF3 low	5	4

p = 0.500

D

	aDTP phenotype	naControl phenotype
ATF3 high	3	0
ATF3 low	0	3

p = 0.050

S5 Fisher exact test of ATF3 and cell death (A), ATF3 and Colonies formation (B), ATF3 and migration (C) and ATF3 and aDTP phenotype (D).

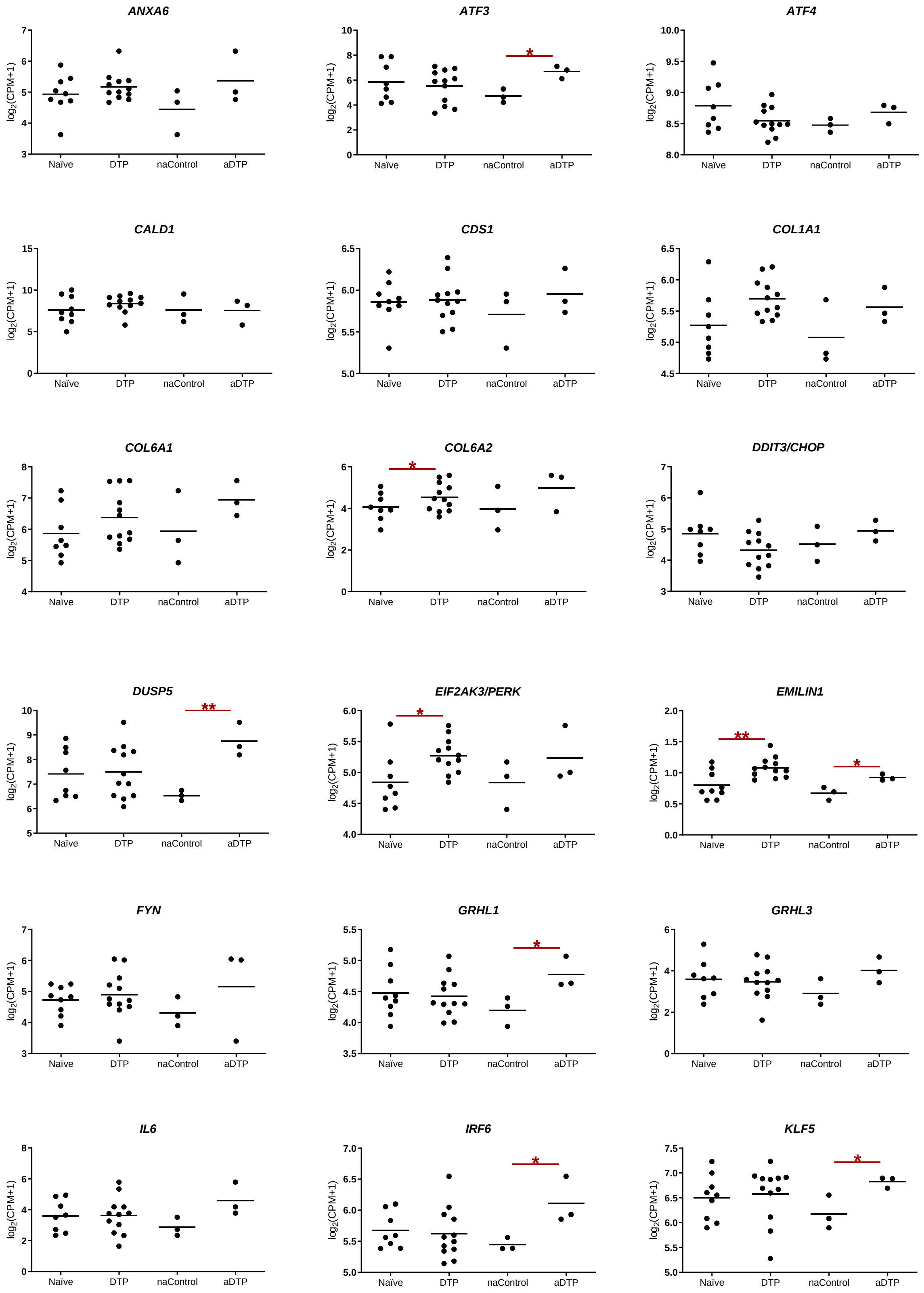
ANXA6
ATF3
ATF4
CALD1
CDS1
COL1A1
COL6A1
COL6A2
DDIT3/CHOP
DUSP5
EIF2AK3/PERK
EMILIN1
FYN
GRHL1
GRHL3
IL6
IRF6
KLF5
log2(CPM+1)
Naïve
DTP
naControl
aDTP
*
**

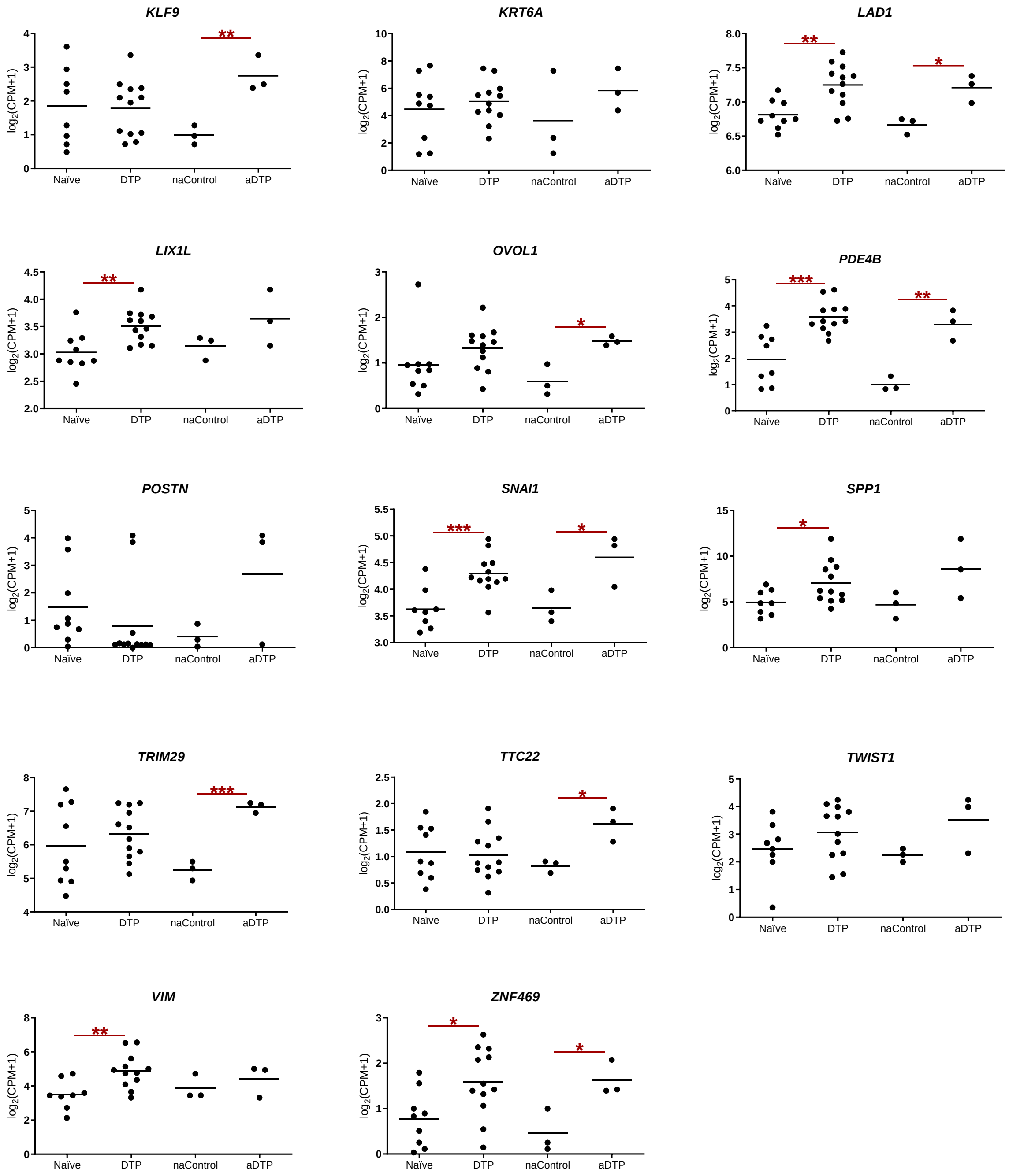

S6 Gene expression of genes from the *ATF3*-associated partial EMT gene signature determined from RNA-seq data: Gene expression of naïve and DTP-derived cells and naControl und aDTP cells are compared. Only significant differences are indicated. *p<0.05; **p<0.01; ***p<0.001

ATF3-associated partial EMT gene signature (partial EMT)		
ANXA6	*EMILIN1*	*OVOL1*
ATF3	*FYN*	*PDE4B*
ATF4	*GRHL1*	*POSTN*
CALD1	*GRHL3*	*SNAI1*
CDS1	*IL6*	*SPP1*
COL1A1	*IRF6*	*TRIM29*
COL6A1	*KLF5*	*TTC22*
COL6A2	*KLF9*	*TWIST1*
DDIT3	*KRT6A*	*VIM*
DUSP5	*LAD1*	*ZNF469*
EIF2AK3	*LIX1L*	

S7 List of 32 stress and EMT-associated strongly differentially expressed genes or genes with a tendency of upregulation in aDTP.

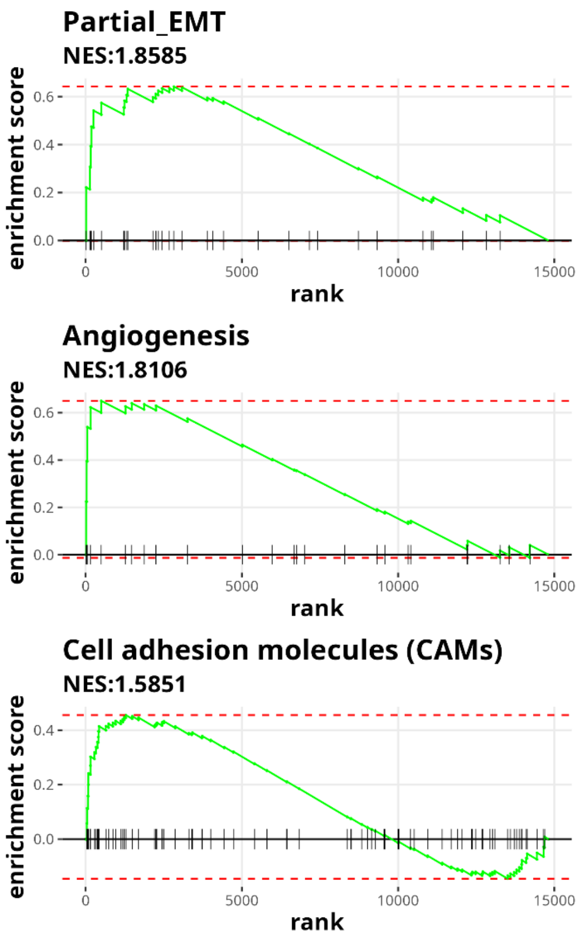

S8 Gene set enrichment analysis of gene expression comparing naïve and DTP clones. Partial EMT, angiogenesis and Cell adhesion Molecules reveal differentially expressed genes that are upregulated in DTPs.

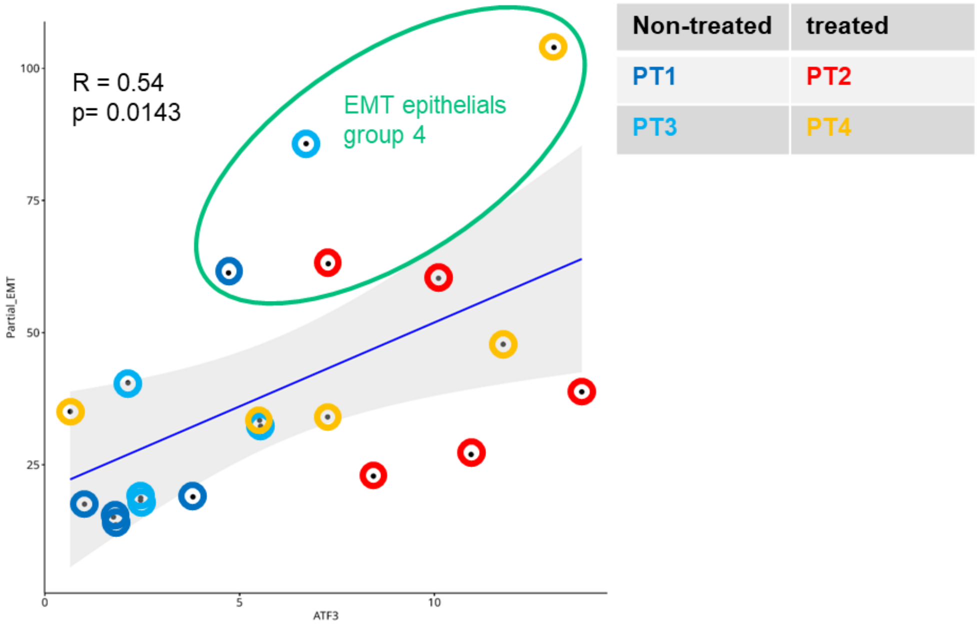

S9 Pearson correlation of 5 epithelial cell groups from four HGSOC patients (Fig. 6H) implies a linear correlation of *ATF3* gene expression to partial EMT gene expression.

S10 *ATF3* gene expression of Epithelial cells from patient 1

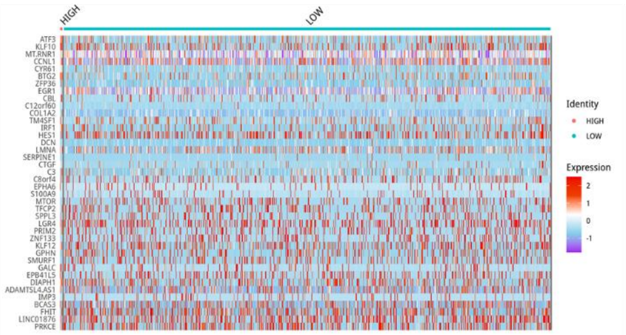

S11 *ATF3* gene expression of Epithelial cells from patient 2

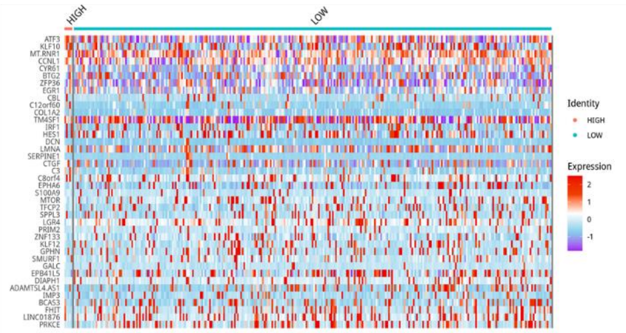

S12 *ATF3* gene expression of Epithelial cells from patient 3

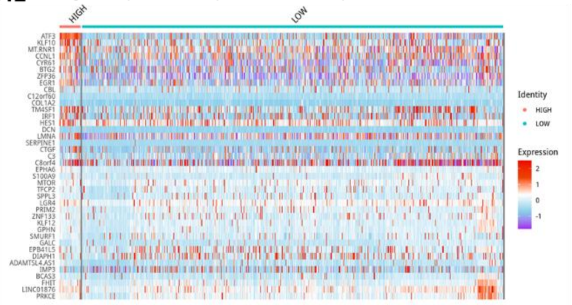

S13 *ATF3* gene expression of Epithelial cells from patient 4

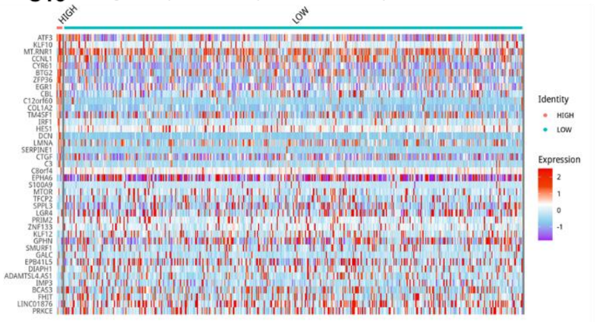

S10-13 Heatmap shows the clustering result of patient material RNA-seq analyzed in single-cell resolution of 4 HGSOC patients. Cells are sorted in high (75% expression) and low *ATF3* expression. Each column represents a cell type and rows represent marker genes. Colors indicate the expression levels as shown in the scale bar. Heatmaps were analyzed together, therefore *ATF3* levels can be compared quantitatively. CPM 75% threshold was defined as high *ATF3* expression.
