# Supplementary figures and images for: ATF3 characterizes aggressive drug-tolerant persister cells in HGSOC
Source: Cell Death Dis. 2024 Apr 24;15(4):290. doi: 10.1038/s41419-024-06674-x (PMC11043376; doi:10.1038/s41419-024-06674-x)

Original Western blot data

Figure 3E

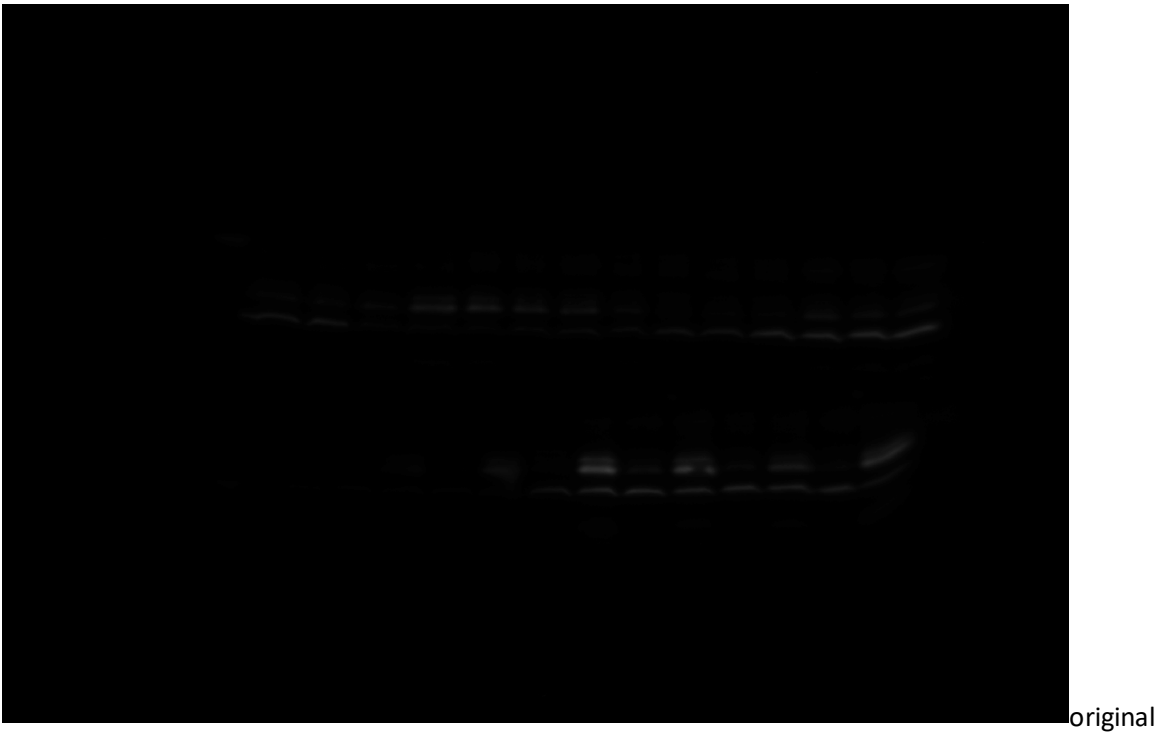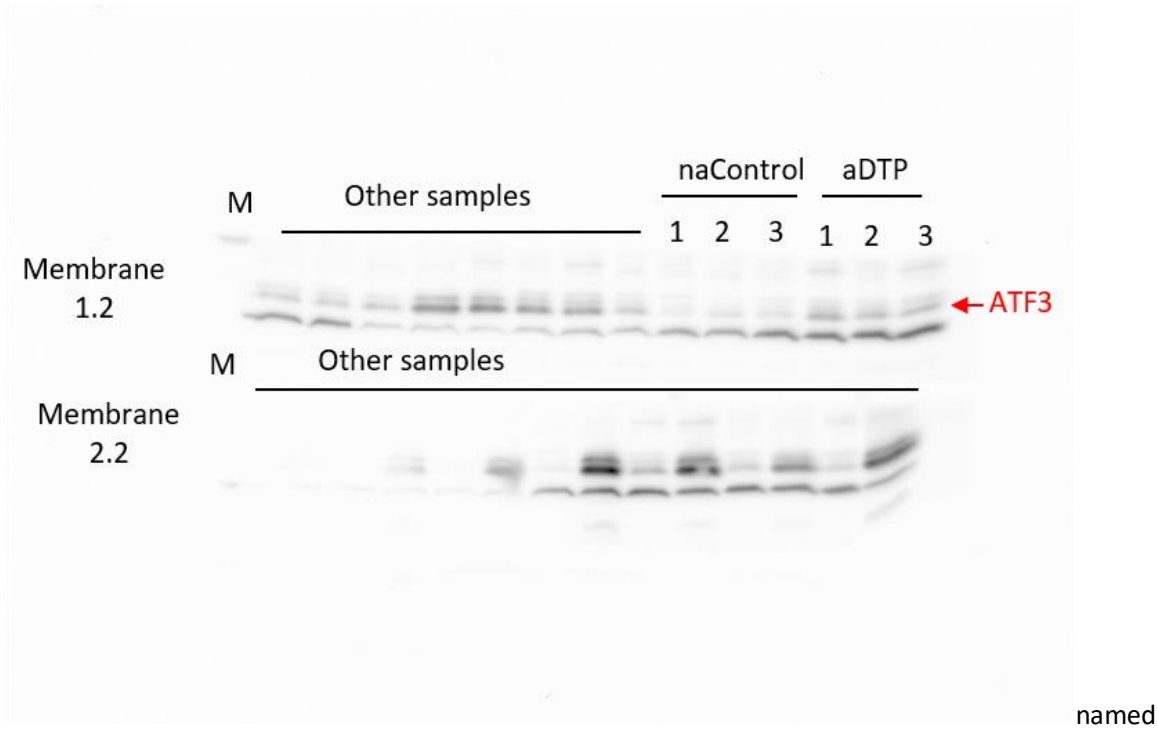

Figure 3F

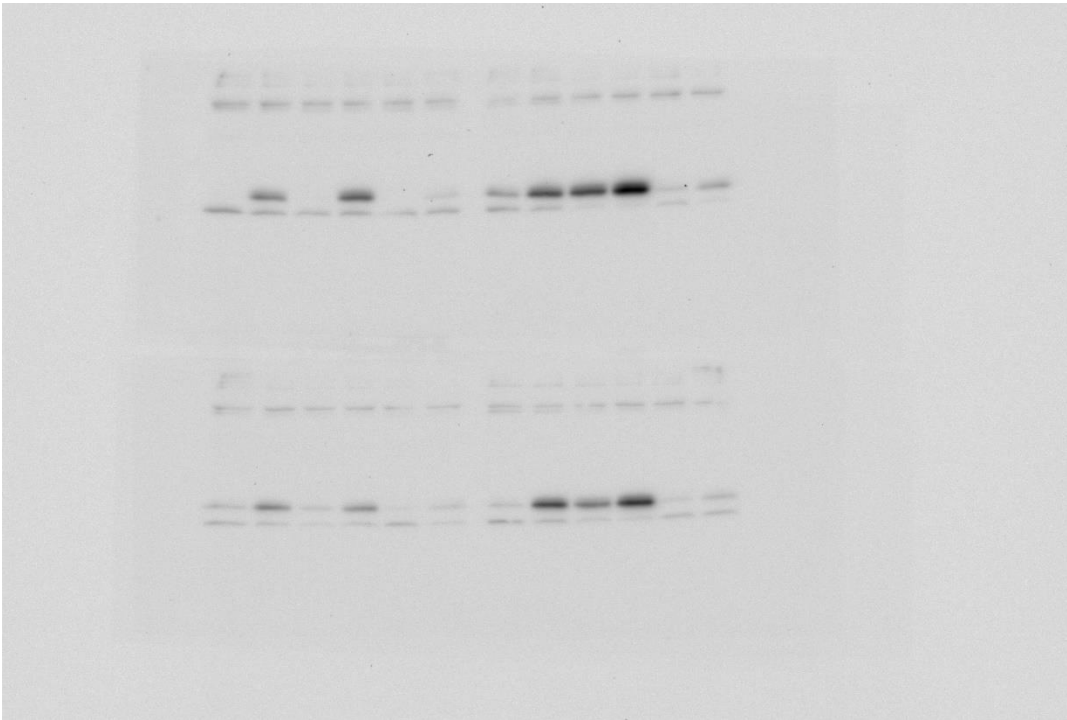

original

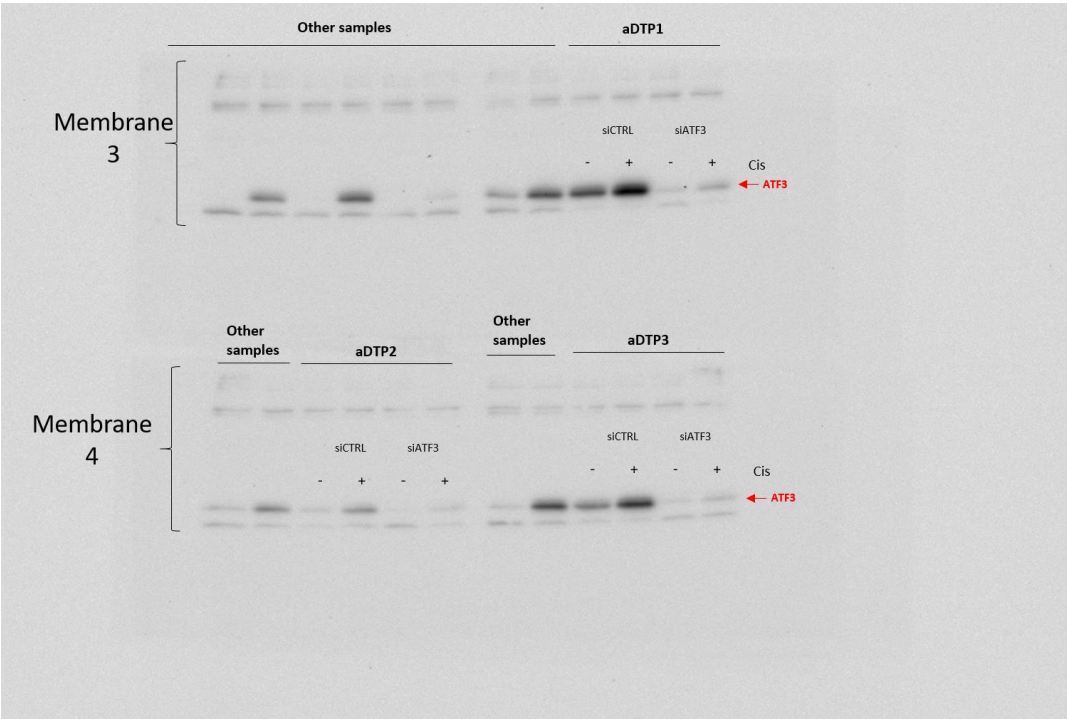

named

Supplement: Supplementary file 2 — Original Data Western blots [file 41419_2024_6674_MOESM2_ESM.pdf]
